# In vitro comparison between inspiration synchronized and continuous vibrating mesh nebulizer during trans-nasal aerosol delivery

**DOI:** 10.1186/s40635-020-0293-7

**Published:** 2020-01-31

**Authors:** Jie Li, Wei Wu, James B. Fink

**Affiliations:** 1 Department of Cardiopulmonary Sciences, Division of Respiratory Care, Rush University Medical Center, 1620 W Harrison St, Tower LL1202, Chicago, IL 60612 USA; 2 Department of Critical Care Medicine, Shanghai Zhongshan Hospital, Fu Dan University, Shanghai, China; 3 Aerogen Pharma Corp, San Mateo, CA USA

**Keywords:** High-flow nasal cannula, Inspiration synchronized nebulizer, Continuous aerosol, Vibrating mesh nebulizer

## Abstract

**Background:**

Compared to continuous vibrating mesh nebulizer (VMN), inspiration synchronized VMN has shown increased inhaled dose during noninvasive ventilation; however, its use during aerosol delivery via high-flow nasal cannula (HFNC) is still unknown.

**Methods:**

An adult manikin was connected to a dual-chamber model lung, which was driven by a critical care ventilator to simulate spontaneous breathing. A HFNC system was utilized with temperature at 37 ° C while gas flow at 5, 10, 20, 40, and 60 L/min. Inspiration synchronized and continuous aerosol generation were compared at different positions (at the inlet of humidifier vs close to patient). One milliliter of albuterol (2.5 mg/mL) was used in each run (*n* = 3). Collection filter was placed at the trachea and was removed after each run. Drug was eluted from the filter and assayed with UV spectrophotometry (276 nm).

**Results:**

When nebulizer was placed close to patient, inhaled dose was higher with inspiration synchronized than continuous aerosol generation at all gas flows (*p* = 0.05) except at 5 L/min. When placed at the inlet of humidifier, compared to continuous, inspiration synchronized aerosol generated higher inhaled dose with gas flow set below 50% of patient inspiratory flow [23.9 (20.6, 28.3)% vs 18.1 (16.7, 19.6)%, *p* < 0.001], but lower inhaled dose with gas flow set above 50% of patient inspiratory flow [3.5 (2.2, 9.3)% vs 9.9 (8.2, 16.4)%, *p* = 0.001]. Regardless of breathing pattern, continuous aerosol delivered greater inhaled dose with nebulizer placed at humidifier than close to patient at all gas flows except at 5 L/min.

**Conclusion:**

When the HFNC gas flow was set higher than 50% of patient inspiratory flow, no significant advantage was found in inspiration synchronized over continuous aerosol. However, inspiration synchronized aerosol generated 30% more inhaled dose than continuous with gas flow set below 50% of patient inspiratory flow, regardless of nebulizer placement. Continuous nebulizer needs to be placed at the inlet of humidifier.

## Background

In recent years, aerosol delivery via high-flow nasal cannula (HFNC) has become a popular measure to deliver aerosol therapy [1–8], particularly for patients who are using HFNC concurrently [1–5]. At regular flow setting, trans-nasal albuterol delivery for stable patients with chronic obstructive airway diseases has been shown to elicit similar response as conventional nebulization treatment [6–8], such as small volume jet nebulizer via mask/mouthpiece or metered dose inhaler with spacer. Similarly, it has demonstrated better tolerance and comfort than jet nebulizer via mask for young pediatric patients [1–3].

Due to its features of no additional gas and little to no residual volume, vibrating mesh nebulizer (VMN) has been commonly utilized in trans-nasal aerosol delivery [1–5, 7–9]. However, the currently available commercial product of VMN generates aerosol continuously. This continuous production causes the waste of aerosolized medication during patient expiratory phase, which is up to three times longer than the inspiratory phase. Because of this drawback, VMN is commonly placed distal from the patient, making the circuit and humidifier chamber act as a reservoir to contain the aerosol generated during expiratory phase, resulting in more aerosol available to the patient [9]. The technique of synchronizing aerosol production with patient’s inspiratory effort is available in small volume jet nebulizer, which shows threefold increase of inhaled aerosol mass over the conventional continuous jet nebulizer [10]. Michotte et al. integrated this algorithm into VMN and compared its efficiency with continuous VMN during noninvasive ventilation; both in vitro and in vivo studies found that inspiration synchronized VMN produced higher lung dosage than continuous VMN [11, 12]. However, little has been known about inspiration synchronized aerosol via HFNC. Thus, our objective of this study was to compare the inhaled dose of aerosol generated by inspiration synchronized vs continuous VMN via adult HFNC.

Gas flow setting during HFNC and patient’s breathing pattern (quiet vs distressed breathing) have been identified as significant influential factors during trans-nasal aerosol delivery [13–18], as such, the two factors as well as nebulizer placement were also compared in our study.

## Methods

### Experiment setup

#### Spontaneous breathing model

**Fig. 1:**
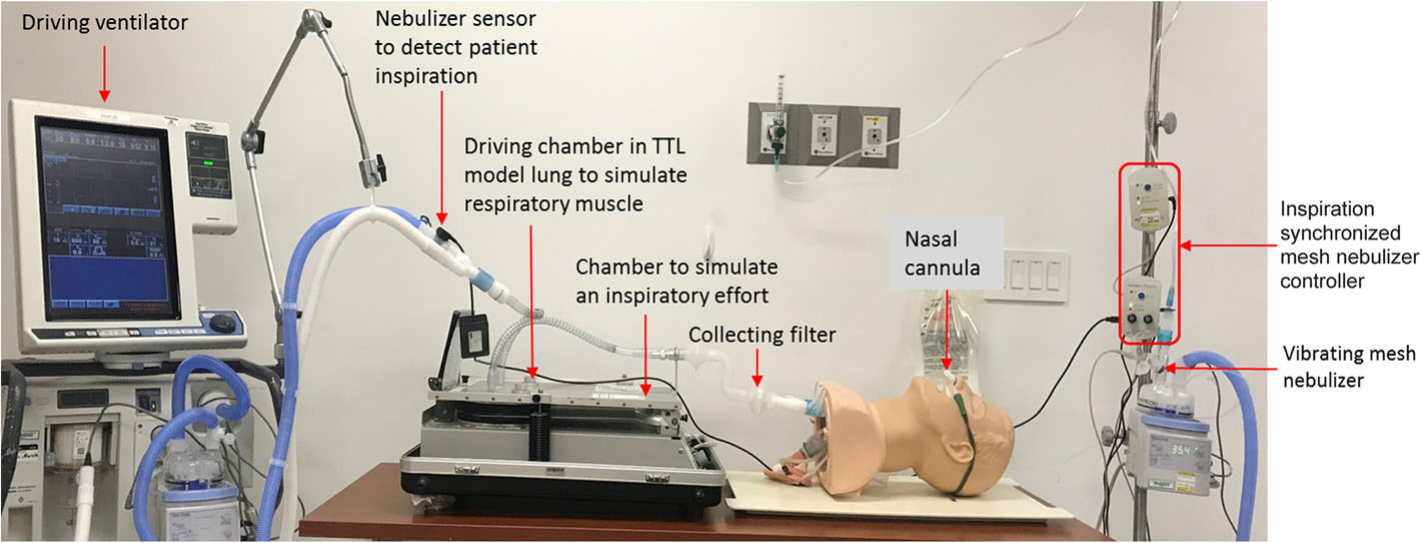
Experiment setup

An adult manikin (adult airway management trainer, Laerdal Medical AS, Stavanger, Norway) with anatomical airway was utilized in this experiment; a collection filter (Respirgard 303, CareFusion, San Diego, CA, USA) which was used to capture the inhaled aerosol was connected with the manikin’s trachea and a dual-chamber model lung (TTL, Michigan Instruments, Grand Rapids, MI, USA). The two chambers could be moved together as for the connection via a rigid metal piece; one chamber was driven by a critical care ventilator (PB 840, Medtronic’s, Minneapolis, MN, USA), functioning as respiratory muscle to move the other chamber which was connected to the manikin to simulate spontaneous breathing. Nasal breathing was simulated by sealing the manikin’s mouth (Fig. 1). Quiet breathing pattern was set as tidal volume (Vt) 500 mL, respiratory rates (RR) 15 breaths per minute, inspiratory time (Ti) 1.33 s, inspiratory to expiratory ratio (I:E) 1:2, and inspiratory flow 22.5 L/min, compared to distressed breathing set at Vt 700 mL, RR 30 breaths per minute, Ti 1.0 s, I:E 1:1, and inspiratory flow 42 L/min [13, 15]. These breathing patterns were achieved by adjusting ventilator settings, with the feedback from measurement by NICO_2_ monitor (Respironics, Murrysville, PA, USA), which was placed between the chamber and trachea.

#### HFNC setup

The Optiflow™ system (Fisher and Paykel, Auckland, New Zealand) was connected to an adult HFNC circuit with a heated humidifier (F&P 850 system, Fisher and Paykel, Auckland, New Zealand); a large size of nasal cannula (Fisher and Paykel, Auckland, New Zealand) was connected with the circuit and placed on the manikin’s nostrils. Humidifier temperature was set at 37 °C. A mass flowmeter (4040, TSI, Shoreview, MN, USA) was utilized to measure and guide accurate flow setting at 5, 10, 20, 40, and 60 L/min.

#### VMN setup

Inspiration synchronized VMN (Aerogen Solo, Aerogen Ltd., Galway, Ireland) was powered by a prototype control module, with a flow sensor placed at the inspiratory limb of the critical ventilator to detect the driving flow simulating spontaneous breathing. Nebulization was initiated when inspiratory flow was detected, and the duration of aerosol spray could be adjusted on the control module. In this study, aerosol spray duration was set at the first 50% of patient’s inspiratory time, which meant aerosol was produced at the beginning 0.7 s of the 1.33 s Ti during quiet breathing and the beginning 0.5 s of the 1 s Ti during distressed breathing. Both inspiration synchronized and continuous VMN were compared at the placement of the inlet of humidifier vs close to patient (between the nasal cannula and circuit). Albuterol powder (1.0 g, Sigma-Aldrich, St. Louis, MO, USA) was reconstituted with 400 mL sterile water to prepare a concentration of 2.5 mg/mL, 1 mL of albuterol was used in each run, and 3 samples were repeated in each experiment.

#### Assay analysis

After each nebulization, the filter was removed and eluted with 10 mL solution (20% ethanol with 0.1 M HCl), which was assayed with UV spectrophotometry (276 nm). After each run, the condensation in the circuit and nasal cannula was emptied and dried. Before initiating each experiment setting, the circuit was stabilized for a minimum of 1 min.

### Statistical analysis

Inhaled dose was calculated as a percentage of the amount of albuterol captured by the collecting filter to the nominal dose (2.5 mg), and expressed as mean ± SD for each experiment setting with different gas flow, breathing pattern, aerosol generation pattern, and nebulizer placement. Mann-Whitney test was used to compare the differences of the inhaled doses with those experiment settings. A *p* value of < 0.05 was considered to be statistically significant. Data analysis was conducted with SPSS statistical software (SPSS 26.0 for Windows; SPSS; Chicago, IL).

## Results

### Inhaled dose of inspiration synchronized vs continuous aerosol via VMN

For continuous aerosol, when nebulizer was placed close to patient, inhaled dose decreased as gas flow increased, regardless of breathing pattern; when nebulizer was placed at the inlet of humidifier, inhaled dose was similar at 5–20 L/min then decreased at 40 and 60 L/min with quiet breathing, while with distressed breathing, inhaled dose increased as gas flow increased from 5 L/min then plateaued at 10–40 L/min. Regardless of breath pattern, inhaled dose was higher with continuous VMN placed at the inlet of humidifier than placed close to patient at all gas flows except at 5 L/min (Table 1).

**Table 1:** Comparison between inspiration synchronized and continuous nebulizer at different placement with quiet vs distressed breathing

Nebulizer position	Breathing pattern	HFNC gas flow rate (L/min)	Inhaled dose (%)	
			Inspiration synchronized nebulizer	Continuous nebulizer
Inlet of humidifier	Quiet (500 mL/15 bpm/1:2/22.5 L/min)	5	19.8 ± .8	19.5 ± .3
		10	27.6 ± 1.2	19.8 ± .8
		20	9.6 ± .3	16.5 ± .8
		40	3.5 ± .1	8.8 ± 1.0
		60	1.1 ± .04	6.3 ± .2
	Distressed (700 mL/30 bpm/1:1/42 L/min)	5	21.0 ± .6	12.9 ± 1.6
		10	23.9 ± 1.3	17.5 ± 1.0
		20	31.2 ± 1.3	17.8 ± .2
		40	9.1 ± .2	16.5 ± .3
		60	2.3 ± .2	9.8 ± .7
Close to patient	Quiet (500 mL/15 bpm/1:2/22.5 L/min)	5	13.4 ± .3	24.1 ± .8
		10	24.1 ± 1.0	15.8 ± .6
		20	18.0 ± 1.8	10.0 ± .4
		40	7.8 ± .6	3.9 ± .3
		60	4.7 ± .4	2.0 ± .1
	Distressed (700 mL/30 bpm/1:1/42 L/min)	5	12.5 ± 1.4	19.0 ± 1.0
		10	24.2 ± .9	17.8 ± .5
		20	23.7 ± .7	13.6 ± .2
		40	12.0 ± .3	8.3 ± .6
		60	6.9 ± .1	3.8 ± .1

**Fig. 2:**
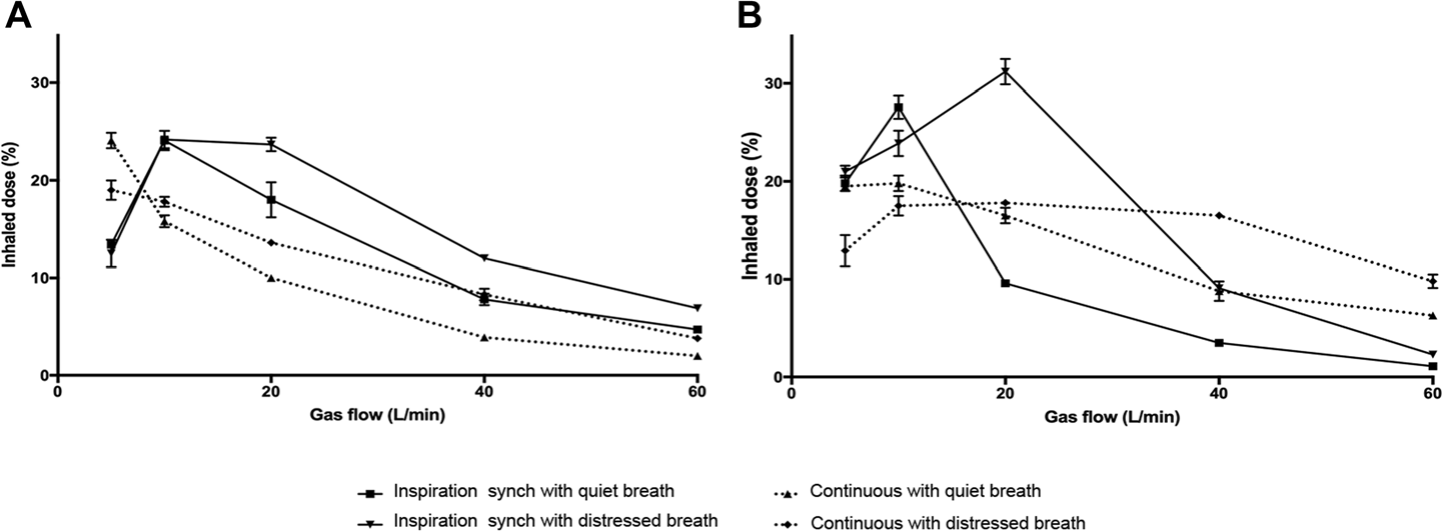
Comparison of inhaled dose (%) between inspiration synchronized and continuous nebulizer placed at the inlet of humidifier (**a**) and close to patient (**b**). Inhaled dose of inspiration synchronized nebulizer peaked at gas flow of 10 L/min during quiet breathing and 20 L/min during distressed breathing, regardless of nebulizer placement. VMN, vibrating mesh nebulizer

For inspiration synchronized aerosol, inhaled dose peaked at 10 L/min with quiet breathing, regardless of VMN placement; while during distressed breathing, inhaled dose peaked at 20 L/min when nebulizer was placed at the inlet of humidifier, and peak inhaled dose was maintained at 10 and 20 L/min when nebulizer was placed close to the patient (Fig. 2).

With nebulizer placed at the inlet of humidifier, continuous aerosol generated higher inhaled dose than inspiration synchronized when gas flow was ≥ 20 L/min in quiet breathing [8.2 (6.3, 16.1)% vs 3.5 (1.2, 9.5)%, *p* = 0.03] and when gas flow was ≥ 40 L/min in distressed breathing (13.2 ± 3.7% vs 5.7 ± 3.7%, *p* = 0.006); however, inspiration synchronized aerosol generated higher inhaled dose than continuous when gas flow was at 10 L/min with quiet breathing (27.6 ± 1.2% vs 19.8 ± 0.8%, *p* = 0.05) and when gas flow was ≤ 20 L/min with distressed breathing (25.3 ± 4.7% vs 16.1 ± 2.6%, *p* < 0.001).

With nebulizer placed close to patient, inhaled dose was higher with inspiration synchronized aerosol than with continuous in both quiet and distressed breathing at each gas flow (*p* = 0.05), except when gas flow was 5 L/min (*p* = 0.33).

### Inhaled dose of inspiration synchronized vs continuous aerosol with the gas flow to patient inspiratory flow ratio

**Fig. 3:**
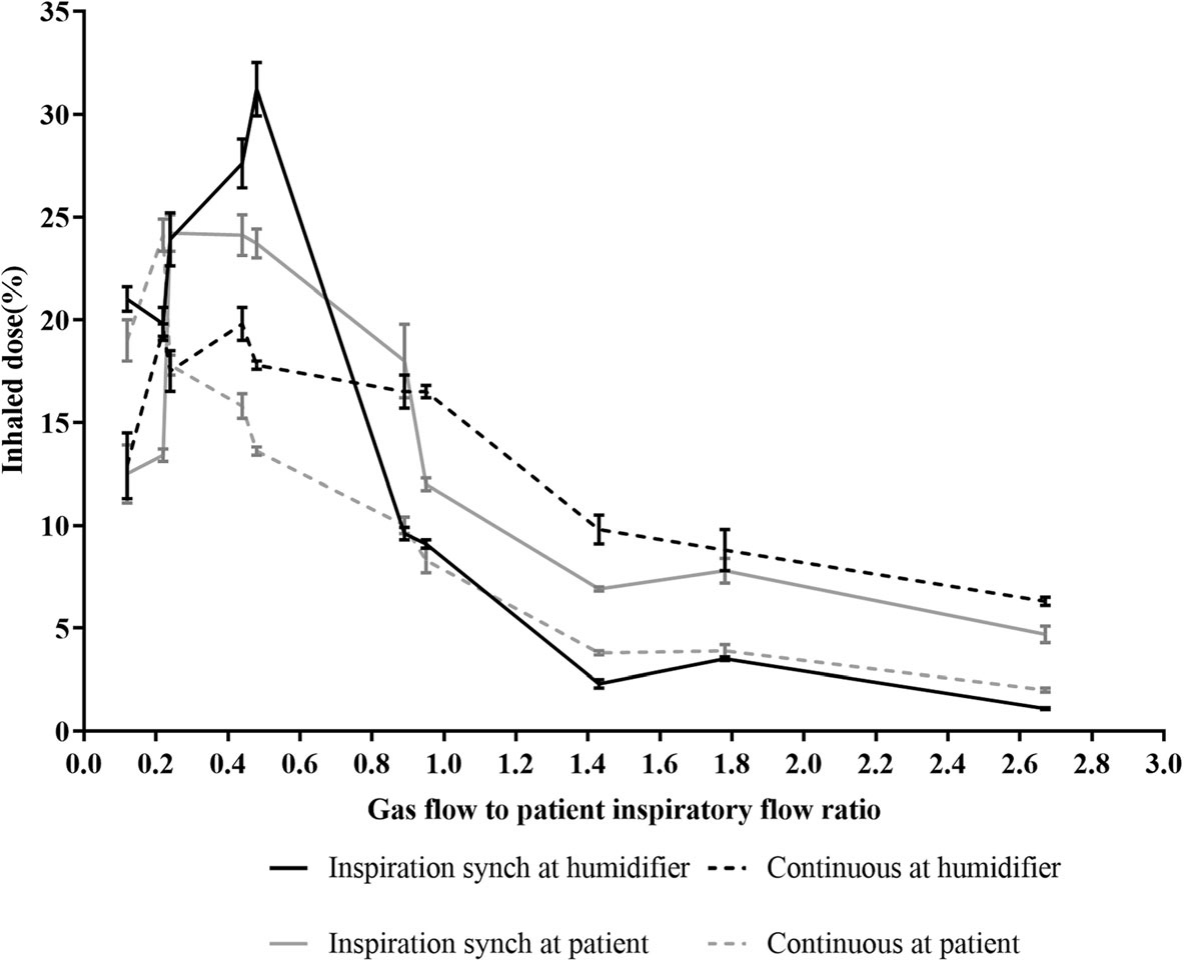
The relationship between inhaled dose and the gas flow to patient inspiratory flow ratio with inspiration synchronized and continuous aerosol

Using the ratio of administered gas flow to patient inspiratory flow (GF:IF) as *X*-axis and inhaled dose (%) as *Y*-axis to draw a scatterplot with inspiration synchronized and continuous VMN placed at different positions (Fig. 3), inhaled dose increased as the GF:IF ratio increased, and peaked around the GF:IF ratio of 0.5, except continuous VMN placed close to patient that peaked at GF:IF = 0.22.

Thus, using GF:IF = 0.5 as a delineator, inhaled dose with GF:IF < 0.5 was higher than that with GF:IF > 0.5 in both inspiration synchronized and continuous aerosol (*p* < 0.001) at both nebulizer placements (*p* < 0.001) (Table 2). When GF:IF was > 0.5, compared to continuous nebulizer, inspiration synchronized nebulizer generated lower inhaled dose [3.5 (2.2, 9.3)% vs 9.9 (8.2, 16.4)%, *p* = 0.001] with nebulizer placed at the inlet of humidifier, but it generated higher inhaled dose [8.0 (6.8, 12.4)% vs 4.0 (3.6, 9.0)%, *p* = 0.010] with nebulizer placed close to patient, and this inhaled dose was similar to continuous nebulizer placed at the inlet of humidifier [8.0 (6.8, 12.4)% vs 9.9 (8.2, 16.4)%, *p* = 0.31]. However, when GF:IF was < 0.5, inspiration synchronized aerosol generated higher inhaled dose than continuous with nebulizer placed at the inlet of humidifier [23.9 (20.6, 28.3)% vs 18.1 (16.7, 19.6)%, *p* < 0.001] and similar inhaled dose with nebulizer placed close to patient (*p* = 0.604).

**Table 2:** Comparison of inspiration synchronized vs continuous nebulizer with different GF:IF and nebulizer placements

Nebulizer placement	Nebulizer type	Inhaled dose (%)		*p*
		GF:IF > 0.5 (*n* = 60)	GF:IF < 0.5 (*n* = 60)	
Inlet of humidifier	Continuous	9.9 (8.2, 16.4)	18.1 (16.7, 19.6)	< 0.001
	Inspiration synchronized	3.5 (2.2, 9.3)	23.9 (20.6, 28.3)	< 0.001
	*p*	0.001	< 0.001	
Close to patient	Continuous	4.0 (3.6, 9.0)	17.6 (15.2, 20.1)	< 0.001
	Inspiration synchronized	8.0 (6.8, 12.4)	23.1 (13.4, 24.1)	< 0.001
	*p*	0.010	0.604	
Comparison of inspiration synchronized nebulizer placed at inlet of humidifier vs close to patient		0.015	0.067	
Comparison of inspiration synchronized and continuous nebulizer at its best position		0.31	< 0.001	

*GF* gas flow, *IF* patient inspiratory flow

## Discussion

To our knowledge, this study for the first time compared the inhaled dose of inspiration synchronized with continuous aerosol generated with VMN at different placements, gas flows, and breathing patterns. When VMN was placed close to patient, inspiration synchronized aerosol generated higher inhaled dose than continuous at all gas flows except at 5 L/min. However, when nebulizer was placed at the inlet of humidifier, compared to continuous, inspiration synchronized aerosol generated lower inhaled dose with GF:IF > 0.5 while it generated higher inhaled dose with GF:IF < 0.5.

### Comparison of inspiration synchronized vs continuous VMN placed at different position

When continuous nebulizer is placed close to patient, little to no aerosol could be stored considering the small volume of nasal cannula; thus, aerosol is largely wasted during exhalation. Inspiration synchronized aerosol generates higher inhaled dose by reducing the waste during exhalation; this explains our findings with nebulizer placed close to patient, which agreed with the finding that higher inhaled dose with breath actuated jet nebulizer than continuous jet nebulizer when it was placed at the Y-piece during invasive ventilation [10]. Moreover, Michotte et al. found higher inhaled dose with inspiration synchronized VMN than continuous VMN during noninvasive ventilation, particularly when nebulizer was placed between the single-limb ventilator and exhalation port; the waste of aerosol was more significant with continuous VMN than inspiration synchronized VMN [11]. In Golshahi et al.’s study, they used similar breathing parameters during quiet breathing (Vt 500 ml, RR 15) but only investigated one gas flow (20 L/min), with nebulizer placed close to patient; they also found higher inhaled dose at nostril level with inspiration synchronized than continuous aerosol [19]. However, we found one exception that with gas flow at 5 L/min, inhaled dose was higher with continuous than inspiration synchronized aerosol, which might be explained by the significant reduction of the washing out effects due to the extremely low flow, resulting in reservoir of aerosol during exhalation for continuous aerosol. In contrast, because of the low flow rate to carry aerosol, aerosol generated at the inspiratory phase might not be able to fully reach the trachea yet before exhalation [20, 21].

Due to the concerns of the aerosol waste during exhalation, continuous VMN was found to generate higher inhaled dose when placed at the inlet of humidifier, using the circuit and humidifier chamber as reservoir during exhalation. However, the reservoir advantages were predominantly with high gas flow, specifically with GF:IF > 0.5. This finding agreed with the results in our previous pediatric study [14]. With GF:IF < 0.5, the reservoir benefits were attenuated; this might be explained by the gravitational sedimentation effects, which increased as the aerosol stayed longer in the circuit and chamber due to the low flow [21, 22]. The optimal period that the aerosol stays in the circuit and chamber to generate the best inhaled dose needs to balance the reservoir benefits and the gravitational sedimentation effects, depending on the reservoir volume and the speed of the transporting gas [21, 22]. As the reservoir volume of adult HFNC system is a constant (~ 1000 mL in adult circuit), this optimal point depends on the transporting gas, including the HFNC gas flow and patient inspiratory flow. From our previous adult in vitro study [13] and this study, using the same adult HFNC system, we found the highest inhaled dose was generated when the GF:IF was around 0.5. The rationale of this number needs more mathematical efforts to explore in the future, but this phenomenon was also seen in both in vitro and in vivo studies with neonatal invasive ventilation that with appropriate reservoir volume and transporting gas flow, continuous VMN even the expiratory actuated VMN could generate higher inhaled dose than inspiratory actuated VMN with inappropriate reservoir volume or transporting gas flow [23, 24]. Similarly, Berlinski et al. speculated that there was an optimal bias flow setting to carry aerosol with continuous VMN placed at the inlet of humidifier during invasive ventilation [21].

In contrast, when inspiration synchronized VMN is placed at the inlet of humidifier, aerosol generated in the inhalation phase might be largely wasted, as patient might already start exhalation when the gas carries aerosol to patient. This asynchrony might be more prominent with high gas flow, which explains our finding that continuous aerosol generated higher inhaled dose than inspiration synchronized aerosol when the VMN was placed at the inlet of humidifier and with high gas flow. However, when gas flow was reduced to < 50% of patient inspiratory flow, this waste was significantly reduced. Instead, aerosol starts to be stored in the system as a bolus before the next inhalation; patient inhales the bolus of aerosol during the next inhalation [22]. Similar to continuous VMN, longer stay with lower transporting flow may cause more gravitational sedimentation; thus, the optimal stay period depends on the HFNC gas flow and patient inspiratory flow, which was found to be approximately 0.5.

In all, when GF:IF was > 0.5, placing inspiration synchronized VMN close to patient generated higher inhaled dose than that at the inlet of humidifier (*p* = 0.010); this inhaled dose was comparable to that with continuous VMN at its best position (inlet of humidifier) (*p* = 0.31). When GF:IF was < 0.5, regardless of the placement, inhaled dose with inspiration synchronized VMN was 30% higher than that with continuous VMN.

### Clinical implication

Compared to the current commercially available continuous VMN, the inspiration synchronized VMN did not generate clinically relevant increment of inhaled dose, particularly at high gas flows. However, this finding was not surprising, as the inhaled dose was only slightly higher with inspiration synchronized VMN than continuous VMN in the previous in vitro and in vivo adult studies during noninvasive ventilation [11, 12], while the inhaled dose was found to be even lower with inspiration synchronized VMN than continuous VMN during neonatal invasive ventilation in both in vitro and in vivo studies [23, 24]. Additionally, all these studies found the delivery time with inspiration synchronized VMN was two- to threefold longer than continuous VMN [11, 12, 23, 24]. As such, the authors argued if it was worthwhile to use inspiration synchronized VMN [11, 12, 23, 24].

Nevertheless, our findings can enhance the understanding of trans-nasal aerosol delivery, which has similarities as well as differences with noninvasive ventilation and invasive ventilation. Particularly, the impact of reservoir benefits vs gravitational sedimentation effects on the trans-nasal aerosol delivery, such as the optimal GF:IF ratio, is worthy to investigate, in order to improve trans-nasal aerosol delivery. Even though no commercially available device allows us to measure patient inspiratory flow breath by breath, the concept of the optimal flow ratio can still guide clinicians to titrate gas flow during aerosol delivery via HFNC, rather than using one constant flow. More importantly, once devices to monitor patient inspiratory flow are commercially available, using inspiration synchronized VMN at the inlet of humidifier at the optimal gas flow can significantly increase inhaled dose by 50–75%. Additionally, regarding the optimal placement of continuous VMN in adult populations, our findings support the current clinical practice to place continuous VMN at the inlet of humidifier rather than close to patient, especially at high gas flow settings.

### Limitations

In this study, we only investigated limited sets of breathing parameters and HFNC gas flows, and only 10 GF:IF ratios were investigated; even the optimal GF:IF (around 0.5) agreed with our previous study [13], it still requires more settings specifically with different GF:IF ratios to confirm. Moreover, we only used one manikin with one size of anatomical airway; even it was made based on the most common size of healthy male adults, our results still could not represent all patients. Future studies with more sizes of anatomical airways particularly with different airway diseases are needed. Additionally, we only investigated the aerosol spray duration at the beginning 50% of inspiratory phase; more studies might be needed to explore different aerosol spray durations to seek for the optimal percentage. Lastly, similar to all the in vitro studies [11, 13–16, 19–22, 24], the manikin’s airway did not have physiological functions such as heat and humidify the gas, filter and absorb the medication by the upper airway, etc; additionally, the filter can capture all the aerosol while some aerosol might be exhaled in vivo, contributing to the higher aerosol deposition in the lung with in vitro study.

## Conclusion

Continuous nebulizer placed at the inlet of humidifier delivered greater inhaled dose than placement at the patient. When HFNC gas flow was higher than 50% of patient inspiratory flow, inspiration synchronized aerosol did not add clinical advantage over continuous nebulizer placed at the inlet of humidifier. However, when the HFNC gas flow was set lower than 50% of patient inspiratory flow, inspiration synchronized nebulizer placed at the inlet of humidifier generated higher inhaled dose than continuous nebulizer, regardless of nebulizer position. Further research is required to understand variables and potential clinical benefits.

## Data Availability

The datasets used and/ or analyzed during the current study are available from the corresponding author on reasonable request.

## Abbreviations

GF:IF: Gas flow to patient inspiratory flow
HFNC: High-flow nasal cannula
I:E: Inspiratory to expiratory ratio
RR: Respiratory rates
Ti: Inspiratory time
VMN: Vibrating mesh nebulizer
Vt: Tidal volume
